
JOURNAL INFORMATION
==============================
NLM Title Abbreviation: Inter Econ
Journal ID: Inter Econ
NLM Journal ID: 101086399

Inter Economics

ISSN: 0020-5346

ARTICLE INFORMATION
==============================
PMCID: PMC7836350
PMID: 33518781
DOI: 10.1007/s10272-021-0941-5
Article ID: 941
Article version: 1
Subjects: Editorial

Restoring Public Trust After Trump and COVID-19

Bourguignon Jiffer j.bourguignon@zbw.eu

Sprenger Ekaterina e.sprenger@zbw.eu

grid.461649.8 0000 0001 1019 0408 Intereconomics, ZBW, Neuer Jungfernstieg 21, 20354 Hamburg, Germany
Electronic publication date: 2021 Jan 26
Print publication date: 2021
Volume: 56
Issue: 1
First page: 2
Last page: 3
Copyright: © The Author(s) 2021
License: Open Access: This article is distributed under the terms of the Creative Commons Attribution 4.0 International License (https://creativecommons.org/licenses/by/4.0/).
Open Access funding provided by ZBW — Leibniz Information Centre for Economics.
License URL: https://creativecommons.org/licenses/by/4.0/

issue-copyright-statement: © The Author(s) 2021

==============================

This was a long time coming. The end of the Trump presidency was shocking but should have come as no surprise. The attack on the US Capitol on 6 January was a fitting, some would even say foreseeable conclusion, to Trump's first and only term in office. After railing for years against the corrupt electoral process that was rigged against him, a majority of the 74 million Americans who voted for Trump believe that the election was "stolen" – despite a lack of evidence, failed legal challenges and refuted claims from election officials in his own party. So when the outgoing president instructed his supporters to converge on Washington DC on 6 January to protest the congressional certification of the Electoral College results that would confirm Joe Biden the next president of the United States, they showed up in droves.

"We will never give up. We will never concede. It doesn't happen", Trump told supporters at his "Save America" rally prior to the attack. "If you don't fight like hell, you're not going to have a country anymore." With this battle cry, he implored them to stream up Pennsylvania Avenue to the Capitol. "I'll be there with you", he assured them.

Over the last four years that Trump has been there with them, his rhetoric and his example have exacerbated already deep divisions, emboldened extremists and encouraged violence. In addition to calling into question the integrity of the US electoral system and refusing to accept the results, Trump has actively tried to disrupt the electoral process by attempting to hobble the US Postal Service; refused on multiple occasions to condemn white supremacists and other hate groups; targeted and banned Muslims from entering the US; separated migrant children from their parents at the southern US border; intimidated, delegitimised and harassed the press – labelling them the enemy of the people; openly called COVID-19 the "China virus" and the "Kung-flu", which in turn resulted in racists attacks against Chinese Americans; politicised a global pandemic by refusing to wear a mask, refuting claims of medical professionals and fomenting discord in cities and states with Democratic leaders who criticised his lack of leadership; used social media to widely disseminate misinformation and conspiracy theories; revelled in lawlessness while insisting upon being the only candidate who would bring law and order during the civil unrest against racial injustice following the death of George Floyd; and – his closing act – incited an insurrection and attack on the very seat of the US government.

The visible symbol of this long but incomprehensive litany of offenses could be seen sleeping on the floor of the US Capitol the week before the inauguration: the National Guard, mobilised to protect the US government from a potential domestic terror attack. Let that sink in.

Since World War II, and perhaps until now, the US has seen its role as one of global leadership: of the defence and promotion of the liberal international order including of democracy, respect for the rule of law and human rights. But during the four years of the Trump administration, the world has witnessed the deterioration of traditional alliances, the withdrawal from international climate and security treaties, the implementation of tariffs and instigation of trade wars and the celebration of authoritarian regimes. The US' standing and reputation has taken a beating internationally. What leg does the US have to stand on in the promotion of its ideals if it cannot rightfully claim to promote and espouse them domestically?

If the US is to continue to play a leadership role on the world stage, President Biden must first exert his leadership on the national stage. The challenges he faces at home are unprecedented in the last half century: reeling in a surging death toll and exploding infection rates during a global pandemic, unemployment and economic stagnation reminiscent of the 1930s, and perhaps the toughest of all, the extreme polarisation of America not seen since the Civil War.

Deep divisions in American society are not new but have as of late been exploited and aggravated by a war on the truth. Without agreement about basic facts, people are disoriented and easily led astray. The evaporation of local news, the rise of infotainment masquerading as news and the prominence of social media have helped to facilitate a parallel universe of alternative facts.

Oxford Dictionaries' word of the year for 2016 was "post-truth", defined as "relating to or denoting circumstances in which objective facts are less influential in shaping public opinion than appeals to emotion and personal belief". Having existed for over a decade, the concept entered the political lexicon and even the mainstream only during the 2016 election campaign of Donald Trump, who does not so much as pretend that objective facts are of any importance and is thus perhaps the best example of a post-truth politician. Across the pond, Brexit was challenging the standards of political communication with its Eurosceptic post-truth rhetoric, along with Grillo's Five Star Movement, Poland's right-wing Law and Justice (PiS) party and Orbán's Fidesz, to name but a few.

Public opinion shaped by emotion rather than evidence and personal beliefs over facts sets identity above policy, creating social conflicts instead of solving them. The erosion of truth goes hand in hand with the erosion of trust, affecting democracies and their institutions. Whether one supports populists or not, they are favoured by voters who perceive the existing political system to be flawed. Post-truth politicians come to power as a response to the failures of democratic governments, governmental institutions and intergovernmental organisations, as low trust in government weakens their authority.

Only a minority of Americans trust the federal government. Around 73% of Americans said they could trust the government always or most of the time when the US National Election Study first asked this question in 1958; since 2014, less than 20% of those surveyed share the same sentiment. The level of public trust is similarly worrying in Europe: trust in the EU's institutions has remained low since it plunged during the financial crisis of 2007-08. According to the most recent Standard Eurobarometer survey, 43% of respondents tend to trust the EU. Trust in national government, however, is gaining ground, albeit on a lower scale, moving up from 36% to 40% since 2019 and ranging from 19% in Bulgaria and 25% in Spain, France and Slovenia to over 70% in Denmark, Luxembourg and the Netherlands.

The COVID-19 crisis is putting people's trust in government to the test yet again. Eurofound's *Living, working and COVID-19* survey published in September 2020 finds that the gap in the level of trust between those who received support during the coronavirus pandemic and those whose requests for support were rejected was approximately 27% for the EU and 43% for the national government. This signals that the coronavirus crisis is exacerbating existing inequalities and has potential impact on social unrest, political disengagement and further polarisation.

Recovery measures and policies and the way these are communicated and implemented will have lasting consequences for public trust. Governments' responses to the economic fallout from the pandemic should be efficient, swift and decisive in order to prevent the coming recession's impact on trust. Rebuilding public trust in a post-truth environment is a challenge not only in Europe, but should be a key priority for the Biden administration in the post-COVID-19 age.

Jiffer Bourguignon, ZBW – Leibniz Information Centre for Economics, Hamburg, Germany.

Ekaterina Sprenger, ZBW – Leibniz Information Centre for Economics, Hamburg, Germany.

